# Holographic Fingerprinting Reveals Oligomer-Driven Phase Separation in Bovine Serum Albumin

**DOI:** 10.1021/cbmi.5c00258

**Published:** 2026-03-27

**Authors:** Fook Chiong Cheong, Seung Y. Lee, Sujata Bais, Satyam Khanal, Saumya Saurabh

**Affiliations:** Department of Chemistry, 5894 New York University, New York, New York 10003, United States

**Keywords:** biomolecular condensates, phase separation, protein oligomerization, Bovine serum albumin, holographic particle characterization

## Abstract

Biomolecular condensates formed through phase separation are fundamental to the cellular organization. Although the physical principles underlying the phase separation of intrinsically disordered proteins are well understood, the molecular determinants of condensate formation in globular proteins remain elusive. Here, we employ holographic particle characterization, a label-free, high-throughput imaging technique, to investigate the self-assembly of bovine serum albumin (BSA), a model globular protein. We show that this technique reliably differentiates between liquid-like and gel-like condensates by their distinct refractive indices and morphologies. Coupled with size-exclusion chromatography, our analysis reveals that BSA phase separation strictly depends on higher-order oligomeric assemblies. Monomeric and dimeric fractions failed to form condensates under identical crowding conditions. Furthermore, the internal packing density of these condensates is tunable via pH-driven protonation changes but remains insensitive to ionic screening. These findings support a model of “emergent multivalency,” in which oligomerization increases the effective interaction valence and creates a structural scaffold that enables hydrophobically stabilized phase separation, consistent with phase separation coupled to percolation frameworks.

## Graphical abstract

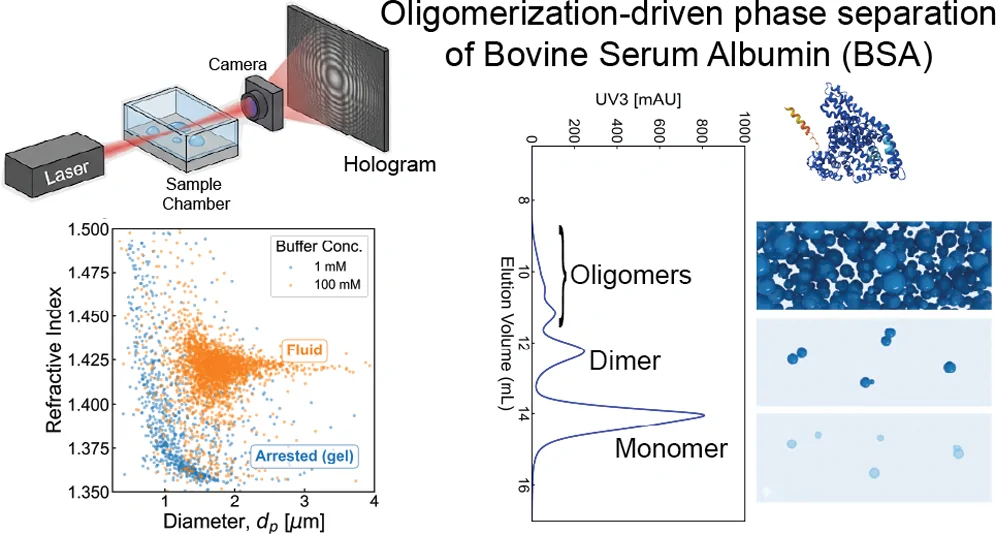

## Introduction

Biomolecular condensates formed via phase separation have emerged as a central organizing principle in cell biology, enabling cells to compartmentalize biochemical reactions without membrane boundaries.^1–3^ Within the prevailing stickers-and-spacers framework, multivalent interaction motifs embedded in structured and intrinsically disordered regions collectively drive demixing once a critical saturation concentration is exceeded.^3–5^ Although this framework was first developed for intrinsically disordered and low-complexity regions, it can be naturally extended to folded proteins by treating surface-exposed interaction patches as “stickers” distributed across the protein surface.^5^ Since many abundant cellular proteins are compact and globular, with limited intrinsic valency and no obvious modular binding domains, fundamental questions remain about how such structured proteins access condensate states in crowded intracellular environments.^6^

Bovine serum albumin (BSA) is a 66.5 kDa globular transport protein and a widely used model for probing the phase behavior of folded proteins under macromolecular crowding. Recent studies have shown that BSA can form micrometer-sized, liquid-like droplets in the presence of polyethylene glycol (PEG) and related cosolutes, with phase behavior tuned by protein concentration, temperature, and solution composition.^7–9^ At the same time, BSA readily undergoes reversible oligomerization into dimers and higher-order assemblies, and can form amorphous aggregates or gels depending on pH, ionic strength, and storage history.^10,11^ This dual propensity for both condensation and aggregation makes BSA an attractive system for disentangling the molecular features that distinguish between condensates with specific viscoelastic properties.^12^

Discriminating between these assembly states remains challenging with standard characterization methods. Bulk turbidity and light-scattering measurements report on overall demixing but cannot reliably differentiate dense liquid droplets from fractal aggregates or quantify their distinct material properties.^13^ Fluorescence microscopy, while powerful for visualizing condensates in cells and in vitro, typically relies on extrinsic labels that may perturb surface charge or hydrophobic interactions, potentially altering the very forces that underlie phase separation, especially for charge-sensitive proteins such as BSA.^14,15^ In contrast, holographic particle characterization uses inline digital holograms to extract both radius and refractive index of individual particles.^16^ This approach has been applied for label-free, high-throughput differentiation of protein aggregates, oil droplets, and other subvisible species based on their optical fingerprints.^17–20^ Recent applications of this technique have demonstrated quantitative sizing and refractive index measurements of protein aggregates and biomolecular condensates, underscoring its potential for dissecting condensate material properties at the single-particle level.^21,22^

Despite growing evidence that BSA can undergo phase separation under PEG-induced crowding, the molecular prerequisites for this transition remain unresolved. It is not clear whether BSA monomers alone are sufficient to drive condensate formation or whether specific oligomeric species act as multivalent scaffolds that license phase separation in analogy to multivalent interaction hubs in intrinsically disordered systems.^23^ Likewise, while crowding and depletion interactions are broadly recognized as drivers of phase separation, the precise crowding thresholds and polymer-size requirements that separate reversible condensate formation from irreversible aggregation in globular proteins have not been systematically mapped.^6,24,25^ Furthermore, the relative contributions of electrostatics, hydrophobic interactions, and protonation state in defining the internal density and material properties of BSA condensates remain poorly understood.^8,26–28^

In this work, we use holographic particle characterization to dissect the phase behavior of BSA in PEG-crowded solutions at the single-particle level. By simultaneously measuring size and refractive index for thousands of assemblies, we establish a robust optical criterion that distinguishes liquid condensates from their gel-like, arrested counterparts and quantify how condensate packing responds to pH, ionic strength, and crowding parameters. Coupling this label-free readout with size-exclusion chromatography (SEC), we show that only higher-order oligomeric BSA fractions generate dense condensates under crowding, whereas monomer- and dimer-enriched fractions remain noncondensing, supporting a model in which oligomerization provides the emergent multivalency required for globular-protein phase separation. Within the phase separation coupled to percolation (PSCP) frameworks, surface-exposed interaction patches on folded domains can also act as “stickers,” and changes in valence or network connectivity can couple percolation to macroscopic demixing.^3,5^ In this view, globular proteins are not intrinsically excluded from phase separation but may require mechanisms that increase their effective valence or promote network formation under crowded conditions. Our findings extend multivalency-based theories of phase separation to structured proteins and highlight inline holographic microscopy as a powerful platform for resolving the molecular determinants of condensate formation and maturation.^4,22,23^

## Results

### Label-Free Detection of Condensates with Distinct Material States

In our previous work, we established holographic particle characterization as a quantitative method to analyze the material properties of condensates formed by the bacterial protein PopZ.^22^ PopZ is an intrinsically disordered protein (IDP) that drives phase separation through flexible, multivalent interactions typical of low-complexity domains. However, globular proteins such as BSA present a fundamentally different physicochemical challenge. Unlike IDPs, globular proteins possess a defined tertiary structure and inherently lack the extended disorder required to facilitate strong, multivalent interactions. Consequently, distinguishing between their arrested or fluid material states is often difficult using conventional bulk assays. We sought to determine if the holographic “optical fingerprinting” we validated with PopZ could be extended to differentiate the distinct assembly states of a globular protein.

To test this, we induced BSA self-assembly in the presence of a crowding agent (PEG 8K) under two distinct buffer conditions: low ionic strength (1 mM sodium phosphate) and physiological ionic strength (100 mM sodium phosphate). Inline holographic characterization resolved two distinct BSA assembly states under crowding conditions that favor condensate formation with distinct material properties (Figure 1). In the 1 mM buffer, BSA formed particulates exhibiting a broad distribution of refractive indices with a characteristic inverse dependence on size: small particulates displayed high refractive indices, whereas larger assemblies showed progressively lower refractive indices (approaching the buffer index). In stark contrast, the 100 mM condition yielded a tight, distinct population of spherical assemblies with consistently high refractive indices (*n*_p_ ≈ 1.40–1.44) that were maintained even as the droplets grew in size.^22^ Although small ions and buffer components may also partition into the dense phase, the much larger refractive index increment of macromolecules compared with ions^22,29,30^ indicates that changes in protein volume fraction are the dominant source of the observed optical contrast.

**Figure 1:**
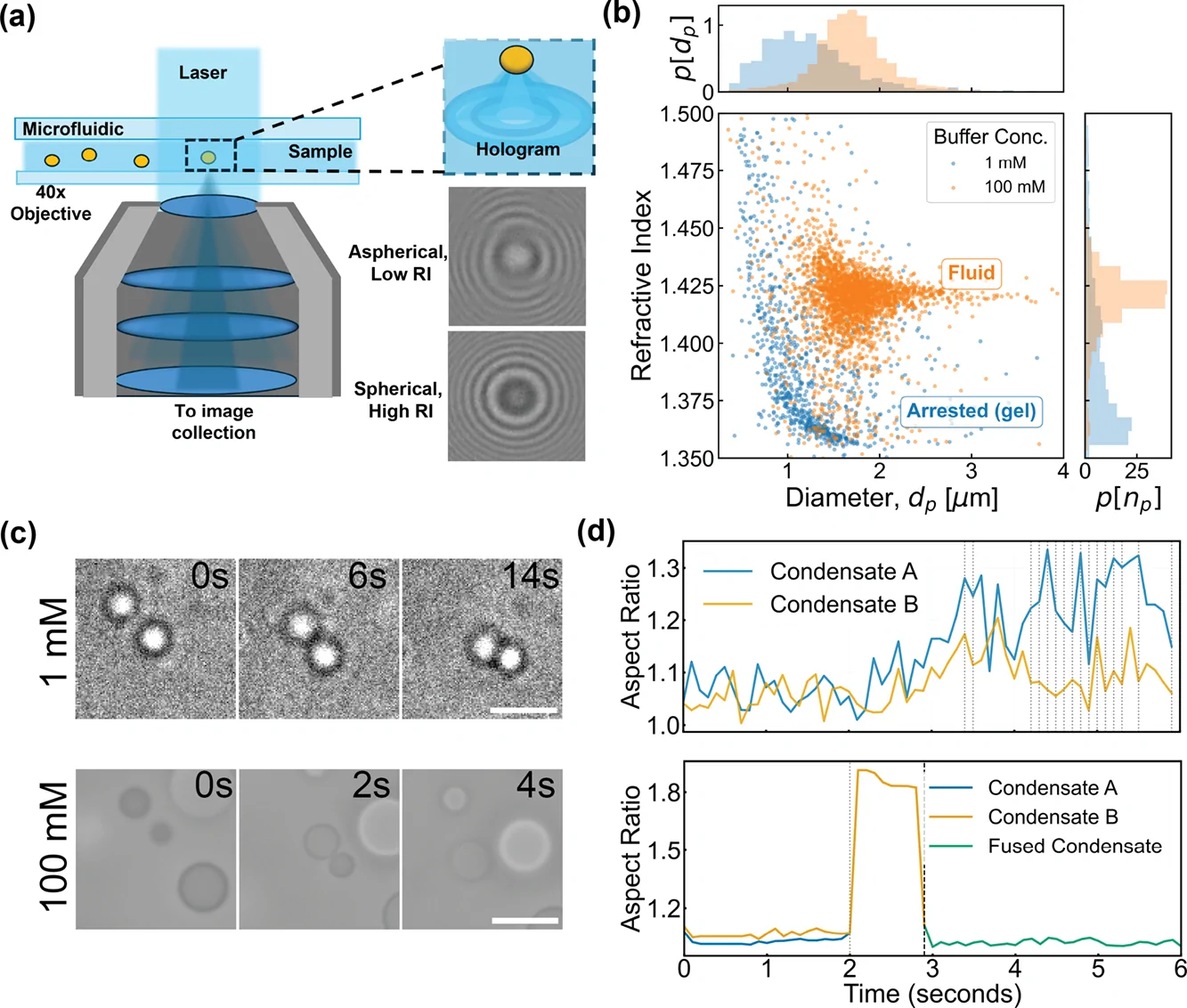
Label-free holographic characterization distinguishes BSA protein condensate material properties. (a) Schematic of the inline holographic particle characterization setup. A laser illuminates the sample in a microfluidic channel, and the interference pattern (hologram) created by the unscattered light and the light scattered by the particle is magnified and recorded. Representative holograms are shown for lower-density (aspherical) and higher-density condensates. (b) Two-dimensional scatter plot of refractive index vs particle diameter (*d*_p_) for BSA assemblies. The plot clearly distinguishes two populations based on the buffer conditions: particles formed in 1 mM sodium phosphate buffer (blue) exhibit a broad size distribution with lower refractive indices, and particles formed in 100 mM sodium phosphate buffer (orange) show a distinct population with significantly higher refractive indices (∼1.40 to 1.44), corresponding to more densely packed biomolecular condensates. (c) Time-lapse brightfield images of BSA assemblies formed in 1 mM (top) and 100 mM (bottom) sodium phosphate buffer under the same conditions as in panel (b). Low-buffer assemblies remain nonfusing and irregular, whereas droplets in 100 mM buffer rapidly fuse and relax to spherical shapes. (d) Aspect ratio traces for individual condensates extracted from the time-lapse movies. Assemblies in 1 mM buffer (top) maintain fluctuating, nonrelaxing shapes, whereas droplets in 100 mM buffer (bottom) show transient aspect ratio spikes during fusion followed by rapid relaxation to ≈1, consistent with liquid-like behavior.

To directly probe the material state under these two buffer conditions, we imaged BSA assemblies by time-lapse bright-field microscopy and quantified their shape dynamics away from the imaging substrate (Figure 1c). In 1 mM phosphate, micron-scale assemblies remained irregular and did not coalesce upon contact over tens of seconds, and their aspect ratios fluctuated without relaxing to unity, consistent with arrested, gel-like condensates rather than a freely flowing liquid. In contrast, in 100 mM phosphate, droplets routinely fused rapidly and relaxed back to spherical shapes, with aspect ratios showing sharp, transient peaks during fusion followed by fast relaxation to ≈1, behavior characteristic of fluid condensates (Figure 1d). These imaging readouts, together with the distinct holographic fingerprints in Figure 1b, indicate that low-buffer conditions favor arrested or gel-like condensates, whereas higher buffer concentrations stabilize a more liquid-like BSA condensate phase.

In this way, single-particle optical fingerprinting, combined with time-lapse imaging, directly differentiates dense fluid condensates and arrested gel-like condensates without labels or staining, overcoming limitations of turbidity assays and conventional imaging in resolving material state and internal density. Together, these data establish that BSA can access multiple condensate material states depending on ionic conditions and that holographic microscopy provides a robust platform for isolating and characterizing these distinct phases for further biophysical analysis.

### Distinct Effects of pH and Ionic Strength on Condensate Density

Having established that BSA condensates represent a distinct, high-density liquid phase, we next investigated the intermolecular forces responsible for maintaining this internal architecture. We asked whether the packing density of these globular protein assemblies could be finely tuned by environmental parameters or if it represents a fixed material state.

We first probed the effect of pH, which directly alters the protonation states of the protein’s surface residues. Tracking the holographic properties of BSA condensates across a pH range of 4.5–7.7 revealed a modest but statistically detectable inverse correlation between pH and condensate optical density (Figure 2a,b). At acidic conditions (pH 4.5), the protonation of acidic side chains brings the protein close to its isoelectric point (pI ≈ 4.7), effectively neutralizing the net surface charge. We hypothesize that this specific protonation state minimizes intermolecular electrostatic repulsion, allowing the globular domains to achieve a closer proximity and higher packing density (*n*_p_ ≈ 1.43). As the pH rises toward physiological conditions (pH 7.0–7.7), deprotonation increases the net negative charge on the protein surface, reintroducing repulsive forces that expand the condensate volume and lower the refractive index to approximately 1.405.

**Figure 2:**
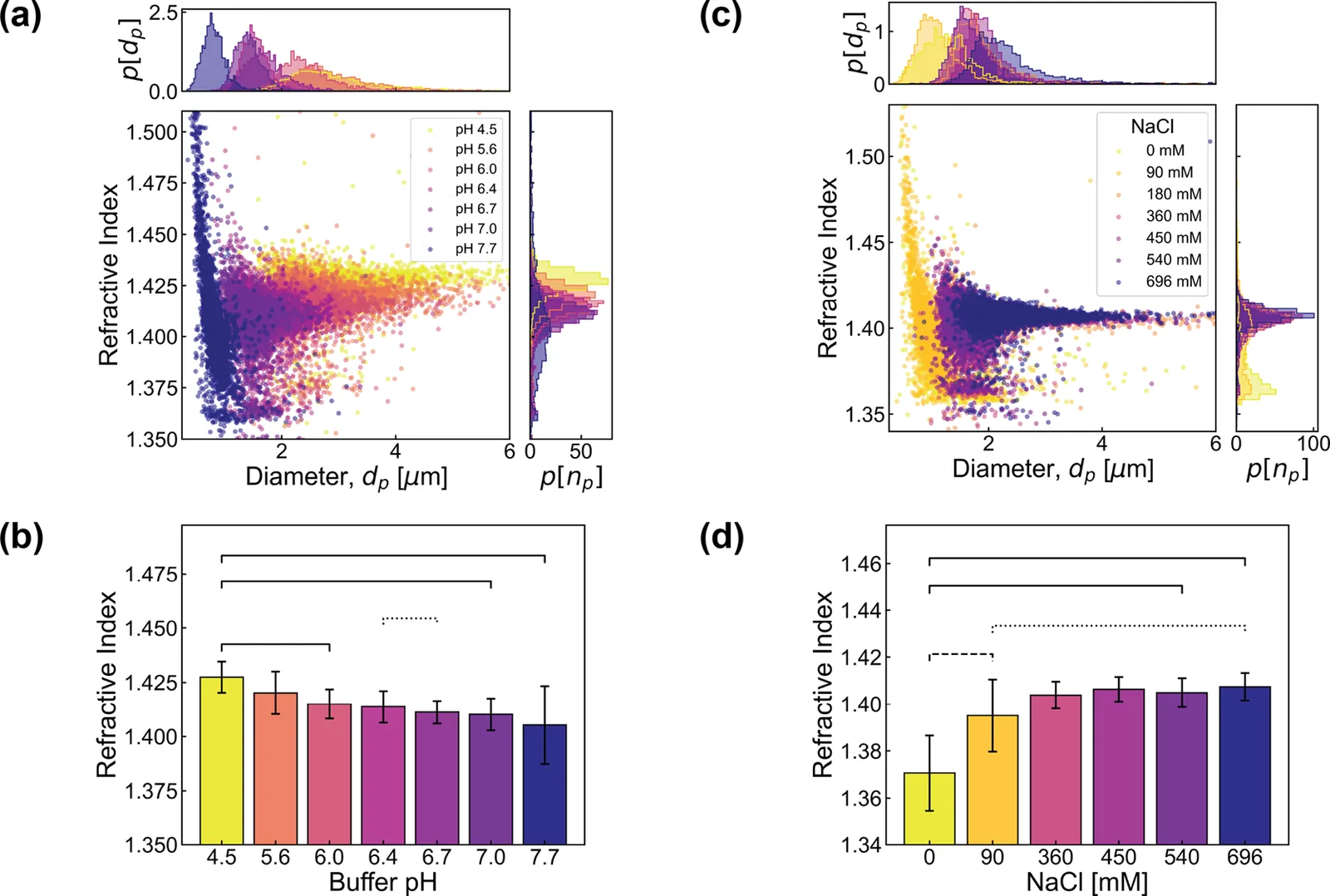
BSA condensate properties are strongly pH-dependent and sensitive within a low ionic strength range. (a) Two-dimensional scatter plots of refractive index vs particle diameter (*d*_p_) for BSA condensates formed at varying pH values (pH 4.5–7.7). (b) Corresponding bar chart of the mean refractive index as a function of pH. Horizontal brackets indicate pairwise comparisons between conditions; line thickness and style reflect the magnitude of Cliff’s delta effect size (|δ| > 0.474, large, solid thick line; 0.330–0.474, medium, dashed line; and 0.147–0.330, small, dotted line). Comparisons with negligible effect size (|δ| < 0.147) are not shown. Adjacent-pair effect sizes are large below pH 6.0 and negligible to small above. The pH-dependent decrease in condensate density is thus driven primarily by the acidic regime and plateaus near neutral pH. (c) Two-dimensional scatter plots of refractive index vs *d*_p_ for condensates formed across a range of NaCl concentrations (0–696 mM). (d) Corresponding bar chart of the mean refractive index as a function of NaCl concentration. The refractive index remains stable at approximately 1.40–1.41 across all salt concentrations tested. This striking insensitivity to a range of ionic strengths suggests that electrostatic interactions are not the primary drivers for maintaining condensate packing density. All condensates were formed in 100 mM sodium phosphate buffer with 20% (w/v) PEG 8K. Error bars in panels (b) and (d) represent the standard deviation of the refractive index distribution.

To quantify the strength of these pH-dependent shifts, we summarized pairwise differences in refractive index using Cliff’s delta, a nonparametric effect-size measure well suited to the non-Gaussian, heavy-tailed single-particle refractive-index distributions obtained from holography. In Figure 2b, large effect sizes are observed between acidic (pH 4.5–5.6) and near-neutral conditions (pH > 6.7), whereas adjacent neutral-pH conditions show only small or negligible effect sizes, indicating that most of the change in condensate density is concentrated in the acidic regime and then plateaus. Taken together, these observations support a picture in which pH primarily tunes condensate packing by modulating surface charge and intermolecular repulsion rather than by changing the underlying cohesive interactions.

We reasoned that if electrostatic repulsion driven by deprotonation regulates packing density, then screening these charges with ions should similarly modulate the material properties. However, when we varied the sodium chloride concentration, the material properties of the condensates displayed no systematic dependence on the ionic environment between 0 and 696 mM salt (Figure 2c,d).^27,28^ The refractive index remained within a narrow range (*n*_p_ ≈ 1.40–1.41) across all salt concentrations, and Cliff’s delta indicated only small effect sizes for all pairwise comparisons. This robustness demonstrates a fundamental distinction in the mechanism of assembly: while the protonation state of specific residues can *tune* the packing fraction by altering the intrinsic charge landscape, bulk ionic screening cannot. This implies that once the optimal protonation state is established, the cohesive forces maintaining the high-density state are predominantly noncovalent and hydrophobic, rendering the internal architecture resistant to simple Debye screening. With the internal density shown to be robust against ionic screening, we turned our attention to the external forces required to drive the initial phase separation, specifically investigating how the excluded volume and depletion forces imposed by the crowding environment modulate the assembly process.

### Critical Crowding Parameters for Phase Separation

While hydrophobic interactions stabilize the interior of the condensate once formed, BSA is intrinsically soluble and does not phase separate spontaneously in buffer alone. This raises a critical question about the nucleation barrier: what are the specific physical parameters of the crowding environment required to force this globular protein into a dense liquid phase rather than an arrested, gel-like condensate?

To define this threshold, we mapped the phase diagram of BSA as a function of the concentration and molecular weight of the crowding agent, poly­(ethylene glycol) (PEG) (Figure 3). We first observed a sharp binary transition based on concentration. At PEG 8K concentrations below 20% (w/v), BSA formed only arrested condensates. A liquid–liquid phase separation regime emerged exclusively at or above 20% (w/v), where stable, high-refractive-index condensates were observed. Furthermore, within this condensed regime, the refractive index increased monotonically with PEG concentration (Figure 3b). We interpret this trend as primarily reflecting stronger depletion and osmotic compression of the protein-rich phase at higher PEG concentrations, consistent with prior work showing that PEG acts largely as an external crowder in protein condensates and partitions only weakly, if at all, into the dense phase.^9,28^ At the same time, because the refractive index reports the bulk optical polarizability of the dense phase, any residual PEG that does partition into condensates will contribute to *n*_p_, and the observed increase in refractive index should therefore be viewed as an effective optical signature of a more crowded, protein-rich interior rather than as a direct measure of protein concentration alone.

**Figure 3:**
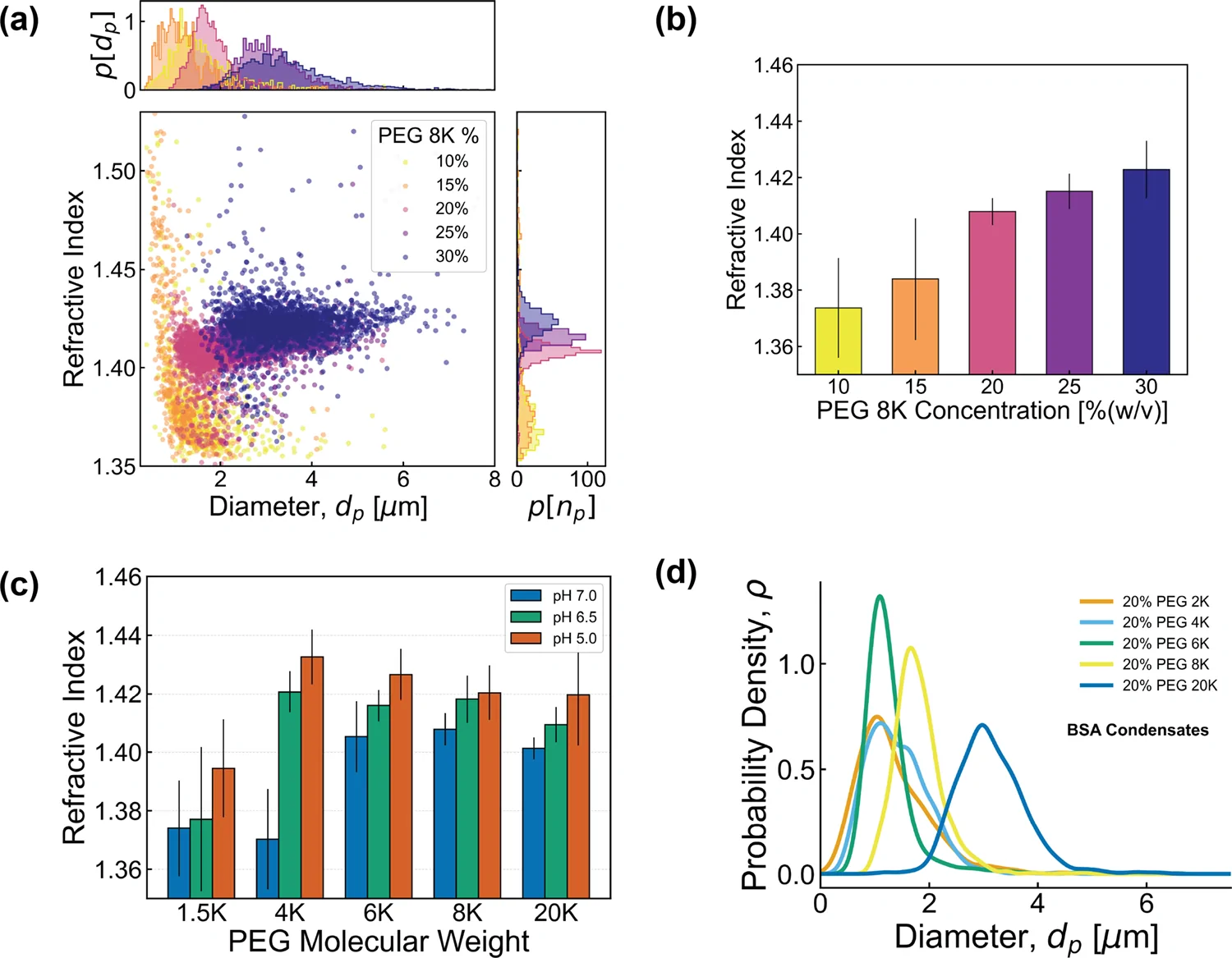
Condensate formation and properties are dependent on the crowding agent. (a) Two-dimensional scatter plots of refractive index vs particle diameter (*d*_p_) for BSA assemblies at varying PEG 8K concentrations (10–30% w/v). (b) Corresponding bar chart showing the mean refractive index increases with PEG 8K concentration. At concentrations below 20% (w/v), BSA primarily forms small condensates with a broad distribution of refractive indices, while condensates exhibiting narrow refractive index distributions are observed at and above 20% (w/v). The increasing refractive index suggests a more densely packed protein environment as the crowding agent concentration increases. (c) Bar chart of mean refractive index as a function of PEG molecular weight (1.5–20 kDa) at pH 5, 6.5, and 7. Condensate formation is dependent on PEG molecular weight; low molecular weight PEGs induce smaller condensates compared to higher molecular weights (>6 kDa) that are required to form dense condensates in a pH-dependent manner. Across all molecular weights tested, lower pH conditions consistently produce condensates with higher refractive indices. (d) Probability density of condensate diameters for BSA samples formed at 20% (w/v) PEG with varying polymer molecular weights (2, 4, 6, 8, and 20 kDa), showing that higher-molecular-weight PEGs promote the formation of fewer, larger condensates, whereas lower-molecular-weight PEGs yield smaller droplets. All experiments were conducted in 100 mM sodium phosphate buffer. Error bars represent the standard deviation of the refractive index distribution.

However, the excluded volume alone is insufficient to explain this behavior. When we varied the polymer chain length while maintaining a constant concentration (20% w/v), we found that molecular weight is a determining factor (Figure 3c,d). Low-molecular-weight PEGs (<6 kDa) failed to induce phase separation, yielding arrested particles, whereas higher-molecular weights (>6 kDa) successfully drove condensate formation. This size-dependence points to a depletion attraction mechanism: the crowding polymer must have a sufficiently large radius of gyration relative to the protein to create an effective depletion zone. Below this threshold, the attractive potential is too weak to sustain the liquid state, and the protein falls into a kinetic trap of arrested particles.

### Oligomerization as a Prerequisite for Condensate Formation

While depletion forces from crowding agents provide the thermodynamic drive for phase separation, they do not explain the molecular specificity of the process. Why do some globular proteins condense, while others aggregate or remain soluble under identical crowding conditions? The current paradigm for protein phase separation relies on “multivalency”—the presence of multiple interaction motifs (“stickers”) connected by flexible linkers. IDPs possess this multivalency intrinsically. Globular proteins such as BSA, however, present a compact, solvent-accessible surface that lacks obvious repeating interaction motifs. We hypothesized that BSA overcomes this limitation not through conformational disorder, but through *oligomerization*, effectively creating a “multivalent scaffold” from multiple globular subunits.

To test this hypothesis, we fractionated BSA using SEC to isolate distinct oligomeric populations (Figure 4a). The elution profile revealed a mixture of monomers, dimers, and higher-order oligomers (likely hexamers and octamers, estimated based on elution volume). We collected individual fractions across this range and subjected them to the exact same crowding conditions established in the previous section (20% PEG 8K, 100 mM NaP).

**Figure 4:**
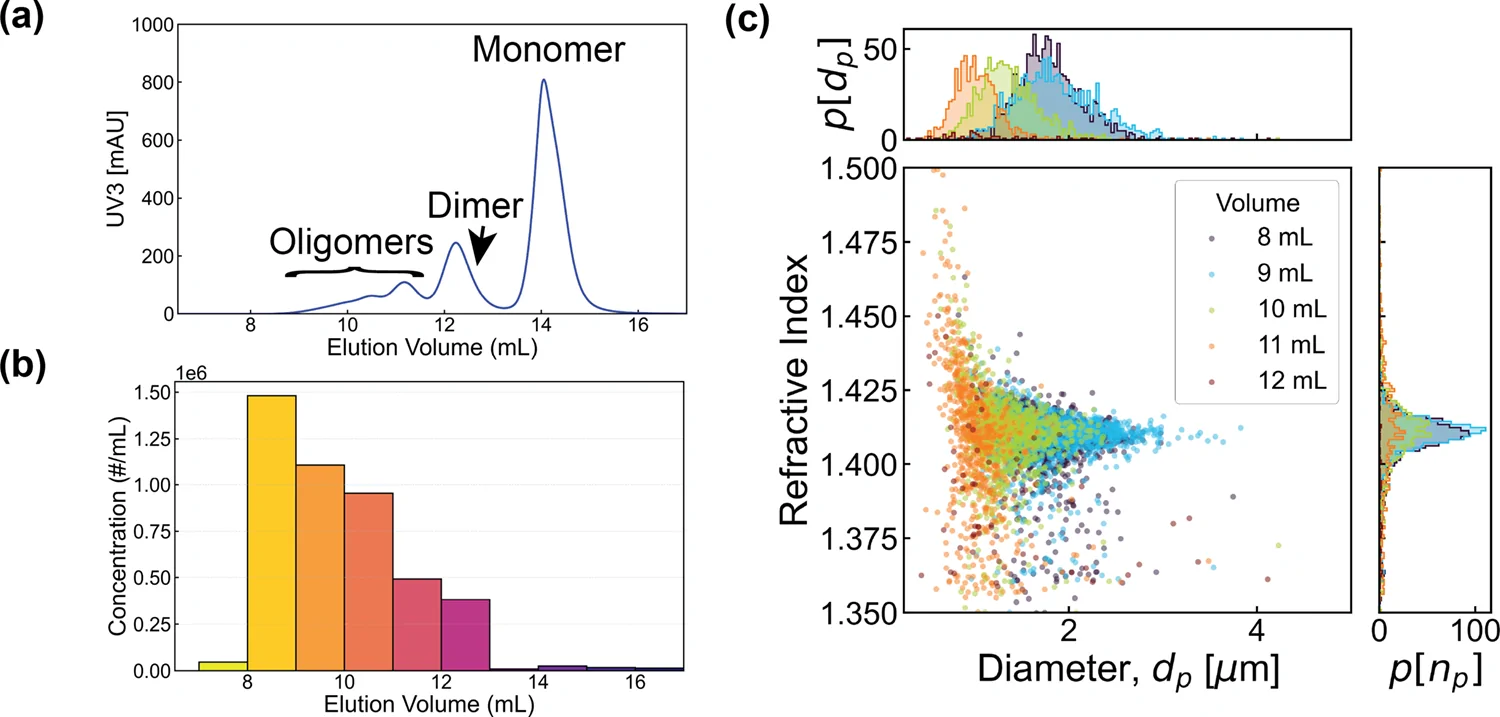
BSA condensate formation is strictly dependent on oligomerization. (a) SEC UV absorbance (UV3 [mAU]) profile of BSA, showing distinct peaks corresponding to monomers, dimers, and higher-order oligomers based on their elution volume (mL). (b) Total particle concentration (measured by holographic characterization) for collected SEC fractions (7–16 mL). The highest particle counts are found in fractions 8–12 mL, corresponding to the higher oligomer and dimer peaks. (c) Two-dimensional scatter plots of refractive index vs particle diameter (*d*_p_) for the corresponding SEC fractions (7–16 mL). The characteristic dense condensate population, identified by a high refractive index (∼1.40–1.42), is present only in the early fractions (7–11 mL) that contain the higher-order oligomers. Later fractions (12–16 mL), which contain primarily dimers and monomers, are devoid of this high-refractive-index population. These results demonstrate that higher-order oligomerization is a prerequisite for BSA to form biomolecular condensates.

The holographic analysis of these fractions provided a definitive molecular criterion for assembly (Figure 4b,c). Fractions containing predominantly monomers and dimers (elution volumes >12 mL) failed to form any detectable high-density condensates; they either remained soluble or formed aspherical, low-index particles. In sharp contrast, the characteristic population of high-refractive-index condensates (*n*_p_ ≈ 1.40–1.42) appeared *exclusively* in the early elution fractions (7–11 mL) enriched with higher-order oligomers.

This result establishes that oligomerization is not merely a side product of crowding but is a strict prerequisite for phase separation in this system. We propose that individual BSA monomers lack sufficient interaction surface area (“stickers”) to support the condensed liquid state. Higher-order oligomerization amplifies the effective valence of the protein complex, enabling it to engage in the cooperative, multivalent interactions necessary to nucleate and sustain a biomolecular condensate.

## Conclusions

The results presented here establish an oligomer-driven mechanism for phase separation of a structured globular protein, extending multivalency-based theories of condensate formation beyond intrinsically disordered systems.^4,22,23^ By combining SEC with holographic characterization, we show that only higher-order BSA oligomers generate dense condensates under conditions where monomers and dimers do not phase separate, indicating that quaternary structure endows a globular protein with emergent multivalency, in line with PSCP frameworks in which increases in effective valence drive both network formation and phase separation.^3^ In our case, oligomerization serves as the percolation-enabling step, converting low-valence monomers and dimers into higher-valence assemblies that can participate in a system-spanning interaction network. This conclusion is consistent with recent work demonstrating that oligomerization domains or coiled-coils can lower condensation thresholds and enable phase separation of modules that do not independently condense.^31–33^

Although our experiments focused on BSA as a model globular protein, we do not expect oligomer-driven multivalency to be unique to this system. Many folded proteins form reversible dimers or higher-order oligomers through defined interfaces, and recent work has shown that oligomerization domains can lower LLPS thresholds or enable phase separation of otherwise noncondensing modules.^28,31–33^ In this context, BSA exemplifies a broader class of globular proteins in which quaternary structure provides the additional “stickers” needed to reach a percolated, phase-separated state, although the applicability of this mechanism will depend on each protein’s oligomerization propensity and interaction geometry. Systematic comparisons across globular proteins with distinct oligomerization behaviors will be required to map how general oligomer-driven LLPS is and where BSA lies within that spectrum.

Figure 5 summarizes a mechanistic model that integrates our observations of oligomer dependence, pH sensitivity, and crowding thresholds into a unified picture. In this model, higher-order oligomers provide the multivalent scaffold required for phase separation, short-range hydrophobic contacts supply the salt-resistant cohesive energy, and the protonation state tunes the condensate mesh size by modulating electrostatic repulsion between oligomeric subunits. The external crowding environment then selects between soluble, aggregated, and condensed states: only when the depletant concentration and polymer size exceed critical values does the system access a stable dense liquid rather than fall into kinetically arrested traps. This framework positions BSA condensates as a globular-protein counterpart to hydrophobically driven phase separation in disordered systems and rationalizes why condensate density is pH-tunable yet largely insensitive to added salt.^27,28,34–36^

**Figure 5:**
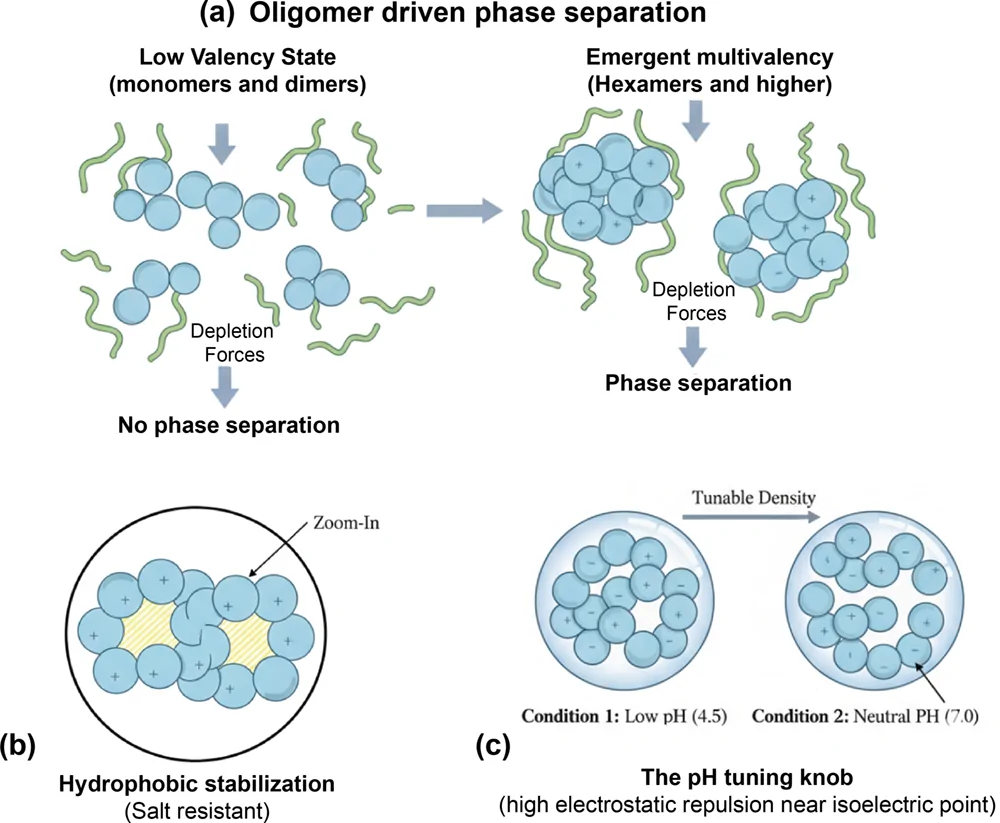
Schematic model for the oligomer-driven LLPS of BSA under crowding. Higher-order BSA oligomers provide the multivalent scaffold required for phase separation, whereas monomers and dimers remain noncondensing or form low-index particles. Short-range, salt-resistant hydrophobic contacts supply the cohesive energy that stabilizes the dense phase, while protonation state tunes condensate mesh size by modulating electrostatic repulsion between oligomeric subunits. The external crowding environment, set by PEG concentration and molecular weight, selects between soluble, arrested, and fluid condensed states, with only sufficiently strong and long-ranged depletion attractions yielding stable, high–refractive-index condensates.

A key advance of this work is the use of inline digital holographic microscopy to distinguish, in a label-free and high-throughput manner, between fluid and gel-like condensates based on their single-particle optical fingerprints. We note that in holographic characterization, the particle refractive index reports the bulk optical polarizability of the dense phase, which depends primarily on macromolecular concentration but can also be influenced by partitioning of buffer components and cosolutes into the condensate. For protein-rich condensates in buffered aqueous solutions, the dominant contribution is generally the protein mass fraction, but potential contributions from small ions and polymers must be considered when interpreting small changes in refractive index.^22,30,37^ By extracting both size and refractive index for thousands of individual assemblies, holographic characterization resolves populations that are indistinguishable by bulk turbidity or conventional imaging and directly links the oligomeric state to condensate material properties. Beyond basic biophysics, misregulated oligomerization and aberrant self-assembly underlie numerous pathologies, including amyloid disorders and aggregation of therapeutic proteins.^38,39^ Because quaternary structure often determines whether proteins remain soluble or form fluid- or gel-like condensates, methods that report on both oligomerization and condensate properties—such as the combination of SEC and holographic particle characterization used here—provide a general framework for dissecting these transitions at the molecular level. More broadly, an oligomer-driven, hydrophobically stabilized mechanism for BSA phase separation suggests that globular proteins can access condensate states without extensive disorder by assembling multivalent scaffolds through oligomerization, complementing models that focus on intrinsically disordered regions and modular interaction domains.^4,22,23^ Practically, the sensitivity of BSA to modest changes in ionic strength, pH, and crowding highlights a narrow energetic balance between physical and chemical interactions, which are directly relevant for protein stability in therapeutic formulations and crowded physiological fluids.^8,26,38^ Future work coupling holographic characterization with perturbations such as chaotropic agents, hydrophobicity modulators, or sequence variants could further dissect the microscopic interactions that govern globular-protein condensates and test whether oligomer-driven multivalency is a widespread strategy for modulating phase separation.^40–44^ Future work will be needed to systematically map how thermal and compositional perturbations drive interconversion between fluid and gel-like condensates in PEG-crowded BSA and to disentangle these pathways from temperature-induced denaturation of the protein. Follow-up studies will be required to determine whether such environmentally tunable phase behaviors are not only biophysically accessible but also crucial for controlling cellular signaling and stress responses, as suggested for bacterial condensates.^45,46^

## Experimental Section

### Reagents

BSA (lyophilized powder, protease-free, MW 66,400 g mol^–1^) was purchased from Tocris Biotechne (EC232-936-2, United Kingdom). Sodium chloride (NaCl) was obtained from Fisher Scientific (S671-3). Poly­(ethylene glycol) (PEG 8000) was purchased from Bioworld (cat. no. 705631) and bioPLUS Fine Research Chemicals. Sodium phosphate monobasic (NaH_2_PO_4_) and sodium phosphate dibasic (Na_2_HPO_4_) were obtained from Sigma-Aldrich (Cat.# S9638 and S9763). All solutions were prepared in deionized water produced by a Milli-Q purification system (18.2 MΩ cm at 24 °C).

### Buffer and Sample Preparation

A 1 M sodium phosphate stock at pH 7.0 was prepared by mixing appropriate volumes of 1 M NaH_2_PO_4_ and 1 M Na_2_HPO_4_ and adjusting the pH with NaOH or HCl as needed. Working buffer solutions at 0.10, 0.010, and 0.0010 M total phosphate concentration were obtained by dilution of the stock with Milli-Q water. Unless otherwise noted, the buffers contained 150 mM NaCl.

NaCl stock solutions were prepared by dissolving NaCl in the corresponding phosphate buffer to the desired concentration and filtering it through a 0.22 μm membrane prior to use.

### Protein Reconstitution

Lyophilized BSA was dissolved in sodium phosphate buffer (pH 7.2, 100 mM total phosphate, 150 mM NaCl) to a nominal concentration of 750 μM. Protein concentration was determined by UV–vis absorption at 280 nm using a NanoDrop spectrophotometer (DeNovix, DS11) and an extinction coefficient of ε_280_ = 44,289 M^–1^ cm^–1^. Samples were stored at 4 °C and used on the same day.

### SEC of BSA Oligomers

BSA oligomeric states were separated by SEC on a Superdex 200 Increase 10/300 GL column (Cytiva, 28-9909-44) equilibrated with 100 mM sodium phosphate buffer (pH 7.0) containing 150 mM NaCl. The column was operated at 0.2 mL min^–1^ on a Biorad FPLC system (Biorad, NGC) at room temperature. After equilibration with at least three column volumes of buffer, BSA samples (e.g., 0.5–1.0 mL at 100–300 μM) were injected, and elution was monitored at 280 nm.

Fractions of 1.0 mL were collected across the elution profile. Oligomeric states (monomer, dimer, and higher-order oligomers) were assigned based on elution volumes relative to a calibration curve obtained using standard proteins of known molecular weight. Protein concentrations in each fraction were determined by UV absorption at 280 nm using the Beer–Lambert law, *c* = *A*/(ε*l*), where *c* is the molar concentration, *A* is the absorbance at 280 nm, *l* is the path length (cm), and ε is the molar extinction coefficient.

### Condensate Formation

For unfractionated BSA, condensates were formed by dissolving 50.0 mg of BSA in 1.0 mL of sodium phosphate buffer (pH 7.0, 100 mM phosphate, and 150 mM NaCl) to yield a stock protein concentration of approximately 750 μM. PEG 8000 was then added from a concentrated stock solution to achieve the desired final concentration (typically 10–30% w/v), and the mixture was gently mixed until the PEG was fully dissolved. Samples were incubated for 15 min at room temperature and then stored at 4 °C until measurement.

For SEC fractions, 70 μL of each protein fraction was mixed with 80 μL of PEG 8,000 solution in the corresponding sodium phosphate buffer to obtain a final PEG concentration of 20% w/v and 150 mM NaCl at the indicated pH values. Samples were incubated at ambient temperature for (10–30) min prior to holographic characterization.

### Condensate Fluidity Characterization by Coalescence

To assess the material state of BSA assemblies, condensate samples were prepared under crowding conditions (20% w/v PEG 8K) in either 100 or 1 mM sodium phosphate buffer and incubated for 5 min at room temperature in an Eppendorf tube. Samples were then transferred to a Tween-20-treated plate for imaging. Condensates were imaged away from the substrate so that the observed coalescence reflected condensate–condensate interactions rather than substrate-mediated effects. Time-lapse brightfield images were acquired on an inverted microscope (Nikon Ti2) equipped with an oil-immersion objective (Nikon PlanApo, 100×, 1.45 NA) and a sCMOS camera (Photometrics Prime 95B) at a system magnification of 0.11 μm per pixel. For each sample, 512 × 512 pixel fields of view were recorded at 100 ms exposure per frame within a 3 min acquisition window.

Coalescence dynamics were quantified from the time-lapse sequences using Fiji (ImageJ)^47^ and custom Python scripts. Condensates formed in 100 mM phosphate were segmented in each frame by Gaussian smoothing (σ = 1.5), Canny edge detection, binary hole-filling, and removal of small objects (<80 pixels), followed by connected-component labeling (scikit-image). Condensates were tracked across frames by nearest-centroid assignment with a maximum linking distance of 20 pixels. Fusion events were identified when two tracked objects merged into a single segmented region, and aspect ratio relaxation of the fused body toward unity confirmed liquid-like behavior. Condensates formed in 1 mM phosphate were segmented by median-filter background subtraction, Gaussian smoothing, and intensity thresholding. Seeded watershed segmentation maintained separate object identities during contact events. Contact between neighboring condensates was detected by dilating one object mask and checking for overlap with the adjacent mask. The aspect ratio of each object was computed as the ratio of the major to minor axis lengths from ellipse fits. Persistent, nonrelaxing aspect ratios upon contact confirmed arrested, gel-like behavior.

### Holographic Particle Characterization

Condensate size and refractive index distributions were measured using an inline holographic particle characterization instrument (xSight, Spheryx Inc., USA). In this approach, samples are flowed through a microfluidic channel (xCell, Spheryx Inc., USA) illuminated by a collimated laser beam (λ = 450 nm). Each particle scatters the incident light, producing an interference pattern (hologram) with the unscattered beam. Holograms are recorded within a viewing volume of approximately 150 × 120 × 50 μm using a 40× objective and a digital camera.

For each measurement, 30 μL of sample was loaded into an xCell device and flowed through the channel at a controlled rate set by the instrument software. Recorded holograms were analyzed using the xSight Total Holographic Characterization software, which fits each hologram to Lorenz–Mie scattering theory to extract the particle’s effective diameter, refractive index, and morphological symmetry at the single-particle level.^48,49^ A typical run lasted ∼15 min, yielding measurements for 10^3^–10^6^ particles in the size range 0.5–10 μm. Particle concentrations were estimated by counting the number of particles within the known sample volume analyzed.

### Statistical Analysis of Refractive Index Distributions

Pairwise effect sizes between experimental conditions were quantified using Cliff’s delta (δ),^50^ a nonparametric measure of the probability that a randomly drawn observation from one group exceeds a randomly drawn observation from another. Effect sizes were classified as large (|δ| > 0.474), medium (0.330–0.474), small (0.147–0.330), or negligible (|δ| < 0.147).^51^ Cliff’s delta was chosen over parametric alternatives (e.g., Cohen’s *d*) because the single-particle refractive index distributions obtained from holographic characterization are non-Gaussian and heavy-tailed, violating the normality and equal-variance assumptions required by *t*-tests and related measures. In addition, with ∼2500 particles per condition, conventional *p*-value tests yield significant results for arbitrarily small differences, and significance thresholds alone cannot distinguish meaningful shifts from trivial ones. As a bounded rank-based statistic, Cliff’s delta does not require equal sample sizes or symmetric distributions, both of which vary across our experimental conditions.
